# Multisectoral approach to achieve canine rabies controlled zone using Intervention Mapping: Preliminary results

**DOI:** 10.1371/journal.pone.0242937

**Published:** 2020-12-01

**Authors:** Vaishali Gautam, Pankaj Bhardwaj, Deepak Saxena, Nitesh Kumar, Dileepan S.

**Affiliations:** 1 Department of Community Medicine and Family Medicine, All India Institute of Medical Sciences (AIIMS), Jodhpur, Rajasthan, India; 2 Department of Community Medicine and Family Medicine, School of Public Health, All India Institute of Medical Sciences (AIIMS), Jodhpur, Rajasthan, India; 3 Department of Epidemiology, Indian Institute of Public Health (IIPHG), Gandhinagar, Gujarat, India; Universidad Nacional Mayor de San Marcos, PERU

## Abstract

**Background:**

Annually, in India, millions of dog bite cases occur; most of them are inflicted by a stray dog. There are over 25 million dogs in the country. The rate of stray dog vaccination is suboptimal in India. This study aims to develop an intervention strategy, using Intervention Mapping framework, tailored for the target community to achieve canine rabies controlled zone.

**Methodology:**

This is an exploratory, cross-sectional study conducted at a tertiary care Medical Institute at Jodhpur, Rajasthan, India, from 2018–2019. The semi-structured, In-Depth Discussion was conducted with a multidisciplinary planning group comprising of members from veterinary, health, and administrative sectors. The In-Depth Discussion focused on knowledge regarding complete stray dog vaccination schedule, self-efficacy (to prevent dog bites), challenges, and barriers faced by residents to achieve canine rabies controlled zone. Further, discussion with veterinary stakeholders focused on challenges faced for rigorous implementation of stray dog vaccination and sterilization.

**Results:**

In-Depth Discussion revealed the following challenges: Lack of participation by the study population for canine vaccination, incomplete knowledge about annual canine vaccination schedule, lack of understanding of dog gestures, lack of infrastructure and resources at veterinary hospitals. The majority of the dogs in the study area were stray dogs that were partially or non-vaccinated and non-sterilized. An intersectoral collaboration was achieved between the community members, veterinary stakeholders both private and Non-Governmental organisations, and heath sector. Following which 35 (76.0%) stray dogs were vaccinated, and 17 (35.4%) were sterilized with community support. Burden of dog bite cases also decreased. The stray dog density map was prepared, and community engagement activity on dog gestures was conducted.

**Conclusion:**

The present study demonstrates the feasibility of achieving canine rabies controlled zone. When implemented in a phase-wise manner across all Medical and Residential complex, this strategy would ensure achieving canine rabies controlled zone through multi-stakeholder engagement.

## Introduction

Rabies is one of the deadliest zoonotic disease, with a 100% fatality rate. Despite preventive measures in place in India, it continues to be a disease of public health concern. Globally, a total of 59,000 deaths are attributable to rabies, of which 95% cases are reported from African and Asian countries [1]. India alone contributes to one-fourth of the global burden [2]. Accurate estimates of deaths due to rabies are unavailable from India as till now rabies is a reportable disease not yet notifiable [3]. Literature has suggested that the burden of rabies deaths in humans and dogs is quite high in India, unlike that reported in the national figures [4,5].

Annually, approximately 15 million dog bite cases occur in India [6]. This considerable burden of dog bite cases is attributable to the stray dog population. There are over 25 million stray dogs in the country. [7]. The primary route of transmission of rabies is via a bite from an infected dog, and considering its high fatality, all cases of dog bites are provided with Post-Exposure Prophylaxis (PEP). Thus, a large stray dog population not only increases the risk of exposure to zoonotic disease like rabies but also imposes a substantial economic burden. The estimated median expenditure for providing Post Exposure Prophylaxis per animal bite victim amounts to $22-$58 [8].

World Health Organisation (WHO) reports that to achieve control of human rabies incidence, it is vital to focus on reducing canine rabies incidence as well [9]. Furthermore, the literature suggests that canine vaccination not only helps to reduce rabies incidence among dogs but is also a cost-effective approach [10–12]. In India, although, National Rabies Control Programme (2012–2017) focuses on preventing rabies among the animal population, especially among stray dogs, but the canine vaccination coverage is far less than the recommended level of at least 70% [3,13].

To meet the global target of eliminating human rabies by 2030 [14], it would be vital to strengthen rabies prevention strategies with multi-pronged efforts focusing not only at the national level but also at the local institutional level. Therefore the current study was conducted with the aim to develop a strategy, using the Intervention Mapping framework, to achieve a canine rabies controlled zone in a tertiary care medical health facility at Jodhpur, Rajasthan.

## Methods

### Study design

This is an exploratory, cross-sectional study conducted at one of the institutes of national importance at Jodhpur, Rajasthan, conducted from 2018–2019, using Intervention Mapping framework. The Institute was established in 2012 and spreads over an area of 67 acres. It includes a hospital complex, a medical Institute building, and a residential complex. The Institute recorded an enormous burden of dog bite cases with 278 dog bite cases reported in 2017–2018 [15]. The stray dogs within the Institute were responsible for the reported dog bite cases. All cases were administered Post-Exposure Prophylaxis. The Institution is an apex body and has laboratory facilities for diagnosing human and canine rabies. From 2018–2019, no human or canine rabies case was diagnosed from the study area.

**Fig 1:**
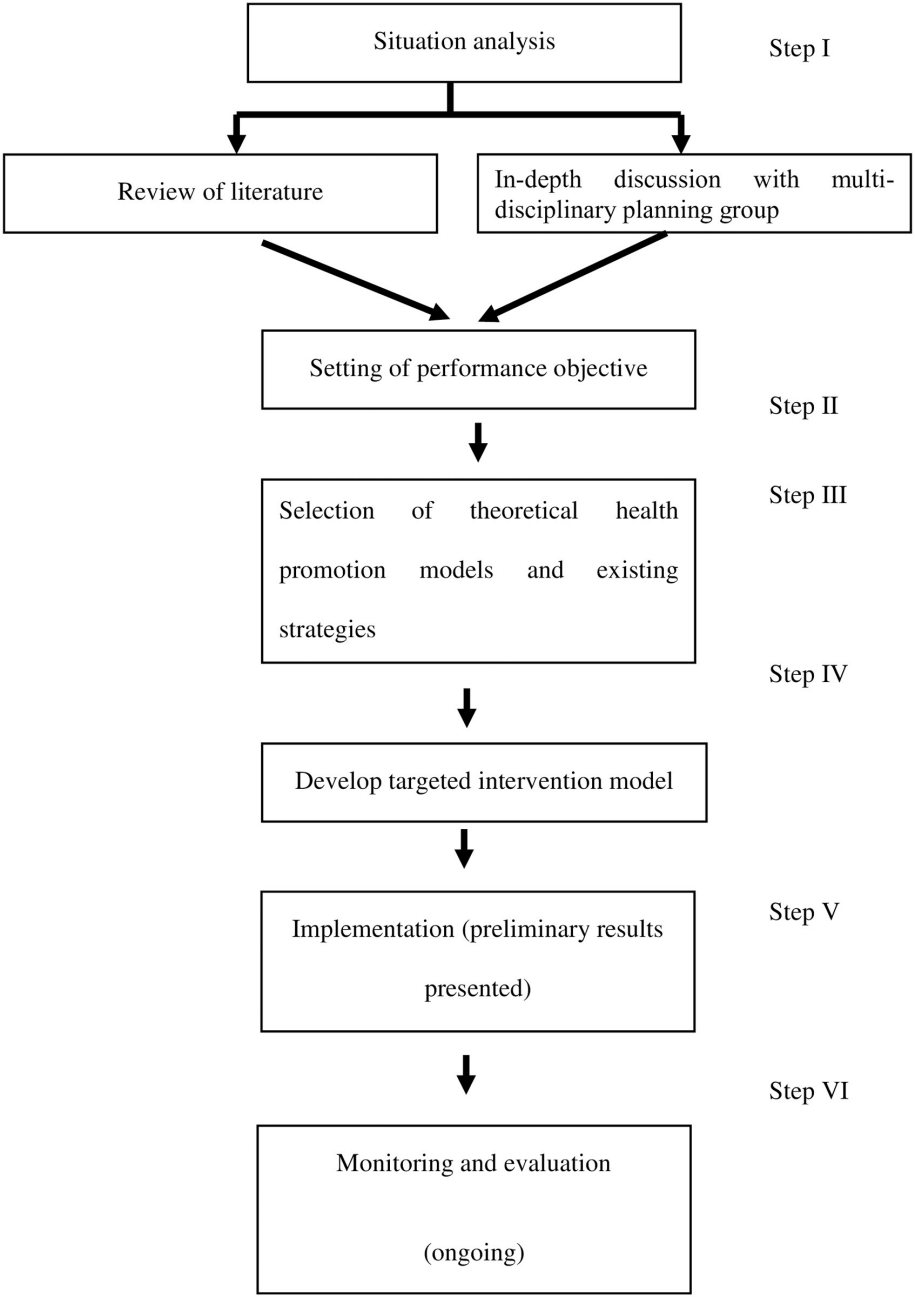
Iterative process of Intervention Mapping adopted in relation to the study. Flowchart representing the Intervention Mapping process.

Intervention Mapping is a systematic process that allows policymakers to develop a health intervention model using scientific evidence, theoretical model(s), and findings from community participation [16]. The six steps of the Intervention Mapping approach, adapted to achieve a canine rabies controlled zone, are depicted in Fig 1.

The present study primarily focuses on conducting step I through IV of the Intervention Mapping framework to develop a strategy to achieve a canine rabies controlled zone. The current study also presents preliminary results of the implementation of the strategy. The quantitative results detailing steps V and VI (implementation and evaluation of the strategy) will be described in the next article.

The first step of Intervention Mapping, i.e., situation analysis which was conducted by performing semi-structured In-Depth Discussion with the multidisciplinary planning group and conducting a scoping review of the literature. Review of literature was conducted to explore factors contributing to rabies virus sustenance in dog populations, resulting in ongoing transmission between dog and human population. Existing intervention strategies to interrupt this transmission and barriers to their implementation were also reviewed.

Multidisciplinary planning group comprised of stakeholders from various sectors. Officials from animal welfare services, state health authorities, and district governing bodies represented the veterinary, health, and administrative sectors, respectively. Representatives from the Institute were also part of the planning group, including residents, children, Institutional staff members, and security staff (representing the health sector and community members from the study area). This group formed the core planning group for the study. All the residents and employees of the Institute were eligible to participate. Children of age ten years and below were excluded as they would be very young to comprehend the information conveyed. The characteristics of the multidisciplinary planning group are detailed in Table 1.

**Table 1:** Characteristics of multidisciplinary planning group.

S.NO.	Planning group members	No. of participants	Relevance of inclusion	Terms of reference
1	Children (5^th^ standard and above) of faculty member and staff members residing in the campus.	8	Community member	Perception about stray dogs within the campus and knowledge about dog gestures.
2	Residents of the Institute (faculty members from various department).	13	Community member	In-Depth Discussion on challenges faced by the campus stray dogs.
3	Undergraduate students (Interns, MBBS and Nursing students) and Postgraduate students.	12	Community member	Discussion on need for adoption of the stray dogs.
4	Security staff members	12	Community member	Discussion on mapping of the stray dog population
5	Municipal cooperation workers	2	Veterinary sector	Experiences about challenges faced regarding canine vaccination and stray dog population management in the district.
6	Non-Government Organisation (NGO) members involved in vaccine procurement (private sector)	2	Veterinary sector	
7	Members from organisation involved in the stray dog handling	2	Veterinary sector	
8	Members of State Health Authorities	2	Health administrative sector	

The discussion focused on different strategies needed to be adopted holistically to successfully develop a strategy to achieve a canine rabies controlled zone. Stray dog population and its associated bite density burden is high in India as well within the study area. Further, the task of rabies vaccination of the stray dogs is mainly passive and dependent upon the participation of Government or local veterinary services, unlike pet dogs, whose ownership lies with its owner. Thus, primary focus was on stray dog population. In this context, detailed description was given regarding the current status of various facilities available at the Institute for rabies control (which is mainly the human component for rabies control), followed by in-depth description of the modalities being implemented at the International and National level. Emphasis was given on canine vaccination and Animal Birth Control for rabies control among the stray dog population.

The purpose of conducting this discussion was (i) To explore the challenges faced due to campus stray dog population, (ii) To initiate a dialogue among the residents to come to a mutual consensus regarding measures which should be adopted to design a canine rabies controlled zone, (iii) Developing a comprehensive dog mediated rabies prevention framework focusing on prevention of rabies in stray dogs.

**Fig 2:**
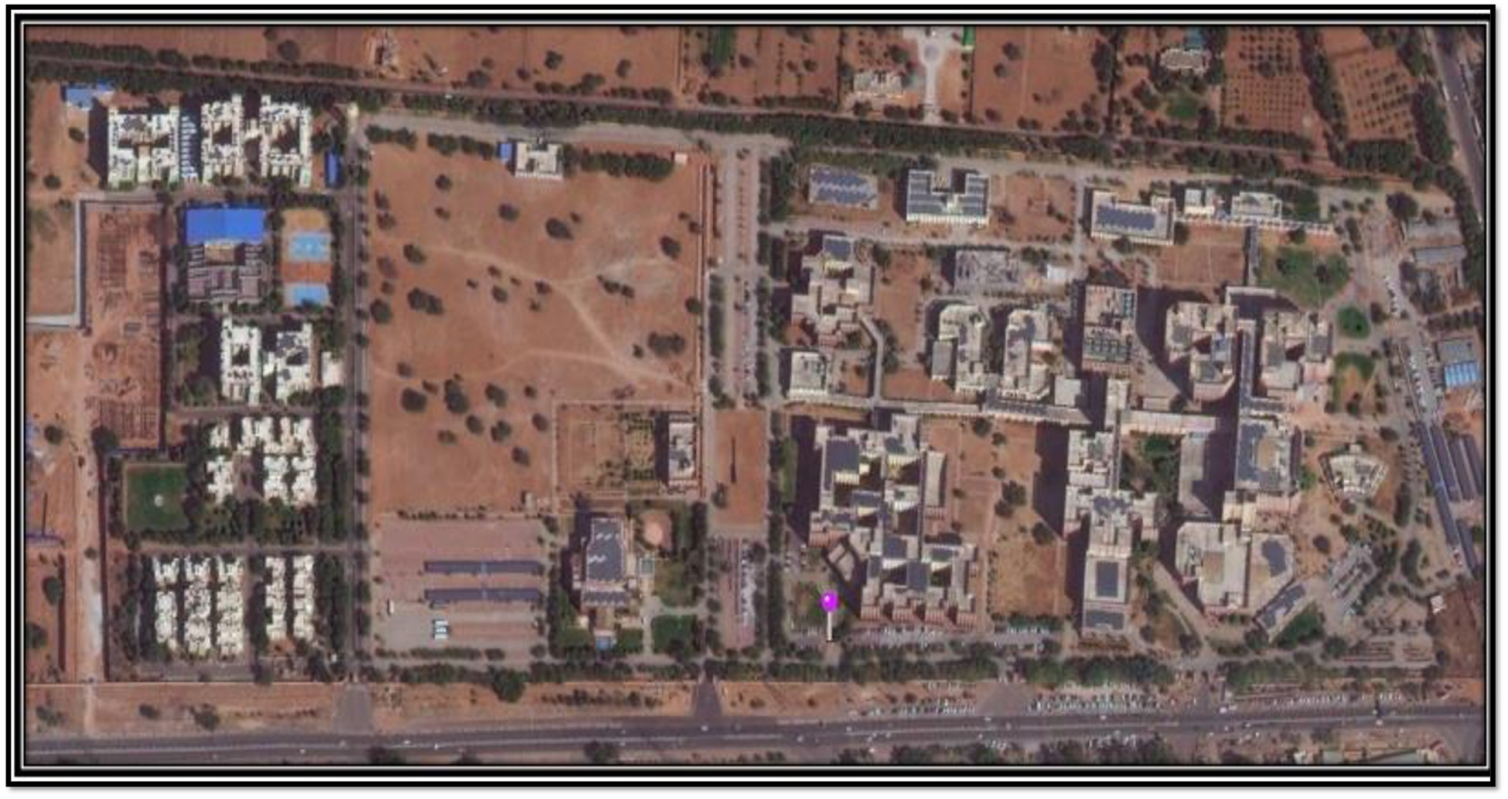
Map of the Institution campus. Map used for stakeholder engagement exercise to indicate stray dog density within the Institution campus.

An In-Depth Discussion was conducted using an interview guide prepared from the findings of the scoping literature review (S1 File). The guide was used to elicit perceptions, beliefs, barriers, and attitudes towards achieving a canine rabies controlled zone. The discussion lasted for about 60 minutes. A stakeholder engagement exercise was also conducted in which the participants were provided with a map of the Institutional campus and were asked to indicate areas of high stray dog density. Shown in Fig 2.

Operational definition of canine rabies controlled zone: It is the zone where both human and canine rabies is notifiable, with surveillance, diagnostic sampling and testing facilities available. In addition, there should be a canine vaccination coverage of 70% and above.

### Data analysis

Notes were prepared after completion of the In-Depth Discussion with the multidisciplinary planning group discussion. Audio recording of the discussion was done. Transcripts were prepared which were reviewed to identify domains by doing manual coding. Thematic analysis was done for semi-structured In-Depth Interview. Themes were developed by two independent teams and then later compared to come to consensus.

### Ethical consideration

The study was conducted after taking approval from the Institutional Ethics Committee. Ethical clearance certificate (certificate reference no. AIIMS/IEC/2019-20/788) was obtained from Institutional Ethics Committee, All India Institute of Medical sciences, Jodhpur. Before implementation of the intervention strategy, due permission due sought from Institution’s Director. Written informed consent was taken from all participants and from parent/guardian of the children.

## Results

### Step I of Intervention Mapping: Needs assessment

Insufficient prioritization of prevention of canine rabies: Veterinary public health services are at a nascent stage in India and needs prioritization [17]. First and foremost, rabies is identified as a reportable disease, but is still not a notifiable disease. Surveillance system for human rabies, indeed is plagued with challenges of under-reporting, inconsistent supply or interrupted administration of PEP, or lack of awareness, but the programmatic emphasis on health education, attempts to identify and overcome logistics challenges are in place and continuously recommended by the researchers as well as Governments officials [18–21].

However, such is not the case when with role of canine rabies vaccination. Sustained canine vaccination coverage have resulted in achieving control of rabies, as exemplified by the developed nations. On account of the high burden of the stray dog population and the enormous burden of dog bite cases, canine vaccination coverage is essential for effective rabies control. Under the National Rabies Control Program, the Animal Birth Control-Anti-Rabies Vaccination (ABC-ARV) strategy is adopted in India [22], where sterilization is followed by the vaccination of the dog.

Routine canine rabies vaccination is recommended but available in an unsystematic manner, only in limited cities, and primarily driven by municipal authorities in the cities with limited funding [23–25]. Thus resulting in inadequate canine vaccination coverage which is reported to be only 15% [13], far less than the desired vaccination coverage of 70% to achieve elimination of canine rabies [26,27]. Even though overall canine vaccination coverage is low, studies have reported that coverage is higher among the owned dogs than among the stray dogs [28, 29]. Thus, the disparity arises where the proportion of stray dogs population and associated bites cases are high, but vaccination is negligible. Furthermore, stray dogs are primarily considered the Government organization’s responsibility and the general population have negative attitude towards them, therefore there is minimal participation of community for adoptions of prevention strategies for rabies control [28,29]. Thus, to comprehensively tackle rabies, prioritization and strengthening of canine rabies prevention efforts is needed with focus on stray dogs, especially in Indian settings.

### Findings from In-Depth Discussion with multidisciplinary planning group

Following domains emerged from In-Depth Discussion with the members of multidisciplinary planning group and the engagement exercise.

#### Institutional residents’ perspectives on achieving canine rabies controlled zone

During the In-Depth Discussion with the Institutional residents to achieve canine rabies controlled zone, it was observed that the stray dog population was the topic of concern.

**Theme 1: Perception regarding stray dog population within the Institute**
Subtheme 1: Apprehension about possible risk of transmission of rabies
  Majority of the residents perceived that stray dogs posed a risk and considered them as “*problematic*” primarily due to fear of rabies. One of the residents shared an experience “*If anyone has seen a case of human rabies*, *the suffering the patients go through*, *we can better understand the threat posed by a dog bite*, *especially that from a stray dog which is unowned*.”
Subtheme 2: Challenges posed by stray dog population
  Anxiety caused due to stray dog population
  Various participants raised the concern for public safety due to unprovoked attacks by the stray dog population. A resident shared, “*I don’t send my kids near the ground*, *rather prefer them to play near house*. *…As they would be away from the dogs near the ground*. *We have seen many dog bites cases in the past*”.
  Another resident concurred and shared, “*My parents who are elderly don’t go for walk on the grounds due to fear of dog bite*. *Sometimes they attack suddenly*”.
  Menace caused by stray dogs
  Some of the participants shared challenges faced due to stray dogs within the campus and stated they are the cause of “*menace”*. One of the participants stated, *“The dogs enter the hallways of residential quarters and defecate thus making hallways unhygienic*.”
Subtheme 3: Stray dog population characteristics
  The campus’s total stray dog population is unknown and was also perceived to be increasing by the Institution residents. Furthermore, it was pointed out that as residential complex gates are open most of the time and it leads to unrestricted entry of new stray dogs into the campus. A resident stated, “*The gates are open most of the time*, *which is the issue of concern as stray dogs outside the campus*, *sometimes enter*. *The guards vigilantly monitor the vehicles’ movement in and out of the campus; why can’t this be for stray dogs also*?”.
Subtheme 4: Preventable burden of bite injuries
  Inadequate understanding of dog gestures: During the discussion, it was revealed that although few dogs bite cases may be considered as unprovoked but the same is not case with all dog bite cases. Some cases occur during feeding the stray dog thus are provoked. Further, it was observed during the discussion that participants lacked knowledge about understanding of the dog gestures. An experience shared by the resident, “*I was bitten by a dog*, *but I had accidentally disturbed him (dog)*, *while he was resting…*. *Such a bite isn’t an unprovoked bite*”.

**Theme 2: Canine rabies vaccination**
Subtheme 1: Facilitators for uptake of Anti-Rabies Vaccine.
  Motivation of the caregivers of stray dogs: It was observed that some residents were motivated and were involved in providing care to the stray dogs. They participated in canine rabies vaccination to prevent rabies and sterilization to address the concern of uncontrolled population growth of stray dog. A resident stated, “*I have taken 10 stray dogs near my residence and got them vaccinated for rabies in the past year*”.
  However, these activities were restricted to their known stray dogs and details of these activities and which dogs are vaccinated were not known to all the residents.
Subtheme 2: Barriers for achieving desired canine vaccination coverage
  Incomplete knowledge on canine vaccination schedule: Residents involved in vaccination of stray dogs had incomplete knowledge regarding complete canine vaccination schedule. Dogs have to be immunised annually to maintain their immunisation status against rabies. This concept was not known to the residents it was the first time they were told about the same.

**Theme 3: Need for institutional administrative support**
Subtheme 1: Intent to adopt a stray dog
  During the discussion it was brought to light that some of the residents had intent to adopt the stray dogs, however as there are no national/local recommendations for the responsible adoption or ownership of stray dogs, therefore they were unable to do so. This was perceived as a threat as it led to confusion on the part of the campus stray dogs’ caregivers.
Subtheme 2: Unregulated stray dog feeding practices
  Stray dog feeding was identified to be a common practice at the residential and hospital campus by the residents. Unmarked sites for stray dog feeding was the primary concern identified. The planning group recognized that feeding a stray dog out of compassion is a humanitarian act, but at the same time, public interest needs to be protected. Regarding this matter, the directions laid down by India’s Animal Welfare Board, 2015, were also discussed [22].
Subtheme 3: Community capacity
  No nets are available with the security staff, nor have they received any skill-based training to securely capture a suspected rabid animal. A resident stated that, “*There was a suspect rabid dog sometime back in the campus*, *no one have standard equipment’s to catch it*”. This case was notified by the Institution to the district veterinary department.

**Theme 4: Need to bridge the communication gap**
It was observed that some residents were involved in vaccination and sterilization of their known stray dogs however, the details were not known to all residents. Thus, there was a need to bridge the communication gap among the residents so that all the residents are aware of these activities.
  After the In-Depth Discussion with the institutional residents, a rapport was built, and it was strongly felt that the stray dogs are the natural cohabitants of the society. Subsequently, all the members could reappraise the situation and acknowledge that the threat of rabies is important and can be manged by canine rabies vaccination. A resident stated, “*It is the first time we have discussed the matter of stray dog population and everyone’s problems*. *We must discuss the issues but*, *at the same time*, *focus on solutions”*.
  A dialogue was also initiated between the members of the residential complex regarding concern associated with stray dogs which led to achieve a healthy consensus that stray dogs are the part of natural ecosystem and, though, they may be the source of zoonotic diseases but if preventive measures are taken like canine vaccination understanding dog gestures, it would greatly reduce the burden canine rabies as well as that of provoked bites occurring within the institutional campus.

#### Veterinary sectors stakeholders’ perspectives on achieving canine rabies controlled zone

**Theme 1: Programmatic implementation challenges**
Subtheme 1: Lack of resources and infrastructure
  The stakeholders involved in veterinary sectors reported that Jodhpur indeed has an ABC-ARV program for control of rabies and dog population management, but its implementation is interrupted due to lack of resources and infrastructure. The supply of Ketamine Hydrochloride (an anesthetic agent) used for animal sterilization at veterinary clinics is inconsistent. Thus, Animal Birth Control activities are often interrupted. Furthermore, veterinary doctors are unable to perform sterilization due to the non-availability of the dedicated operation theatre. On this, an Institution resident stated, “*I went to the hospital to get my known stray dog sterilized*, *but the service was refused on account of lack of Ketamine Hydrochloride*. *As health Institutions*, *we have adequate supply of Ketamine Hydrochloride and can collaborate with the veterinary sector to support their activities*.”
Subtheme 2: Fallacious views of general population
  During the discussion, one of the reasons stated for the low uptake of dogs’ sterilization activity was the general perception of the population that ABC activities would eliminate the dog population. A stakeholder shared an experience “*Even when we have resources*, *the program’s ABC component has low uptake mainly due to fear of elimination of dog population*.”

**Theme 2: Need to collaborate with community and veterinary health sector**
The planning group perceived that there is an urgent need to address the situation of unvaccinated and unsterilized stray dogs of the Institutional campus to achieve canine rabies controlled zone. Although, caregivers are participating in the canine rabies vaccination, the activity needs to be implemented for all dogs, especially for the campus’s stray dogs. It was strongly perceived by the resident that the recommended vaccination coverage level should be achieved along with yearly vaccination of the stray dogs to maintain their (dog) immunization status.
  The Institutional residents acknowledged that the desired goal can be achieved by participation of more community members along with support of the veterinary department. Thus, coordination between the community and veterinary department was needed for sustainable results and to successfully conduct annual vaccination for stray dogs.
  Subsequent to discussion Institutional residents came forward to take responsibility for motivation and initiation of rabies vaccination of the campus stray dogs.

Results of thematic analysis; themes, subthemes and area explored are depicted in Table 2.

**Table 2:** Themes and subthemes generated subsequent to thematic analysis.

Area explored	Themes	Sub-themes
Institutional residents’ perspectives on achieving canine rabies controlled zone	Stray dog population	Apprehension about possible risk of rabies transmission Challenges posed by stray dog population Stray dog population density Preventable burden of bite injuries
	Canine rabies vaccination	Facilitators for uptake of Anti-Rabies Vaccine Barriers for achieving canine vaccine coverage
	Need for Institutional administrative support	Intent to adopt the stray dog Unregulated stray dog feeding practices Community capacity
	Need to bridge the communication gap	Developing a healthy relationship and rapport between the residents
Veterinary sectors stakeholders’ perspectives on achieving canine rabies controlled zone	Programmatic implementation challenges	Lack of resources and infrastructure
	Need to collaborate with veterinary health sector	Coordination between community members and veterinary stakeholders

### Step II of Intervention Mapping: Setting performance objectives

Quantifiable performance and change objectives were defined to achieve the goal of achieving a canine rabies controlled zone. Canine rabies vaccination, prevention of provoked dog bites, and campus stray dog population control were targeted by focusing on achieving change in the participant’s health-related behavior and modifying the environmental determinants. Change objectives describe the behavioral and environmental changes required to achieve the defined outcomes. The target population for behavioral change were the institutional residents. The institution’s administrative body and residents were approached to address the environmental changes. Depicted in Table 3.

**Table 3:** Setting of performance and change objectives.

	Performance objectives	Change objectives
1.	To ensure canine rabies vaccination and microbiological testing for rabies	Identify the caregiver of stray dogs within the campus and constitute stray dog welfare committee. All residents should facilitate and support the stray dog welfare committee in annual stray dog vaccination. All security staff should perceive themselves as skilled to assist in the safe capturing of a possibly rabid dog. The availability of dog entrapping nets should be ensured. Maintain coordination with the veterinary hospital and stakeholders involved in vaccine procurement (private sector) to ensure continuity of canine rabies vaccination at the institute. Identify the campus dogs which are unvaccinated and communicate the same to the dog welfare committee of the institute. Regular appraisal canine rabies vaccination to address the challenges faced. Ensure the functioning of the institute’s microbiology laboratory (for conducting microbiological diagnosis for rabies for both humans and dogs). Ensure the functioning of an integrated surveillance system for possible rabid animals. Ensure a continuous supply of Post-Exposure Prophylaxis in the event of a dog bite. In situation when a case of human or canine rabies is diagnosed, it would be notified to district, state and national authorities.
2.	To control stray dog population and prevent unprovoked dog bites	Maintenance of stray dog population registry. Estimate annual burden of dog bites and document whether it was provoked or unprovoked bite. Resident should feel confident in understanding dog gestures. Restriction of entry to any new dogs in the zone by vigilant guarding of the gate by the guards. Residents should be able to understand the need for stray dog population control. Animal birth control activity conducted by stray dog welfare committee should be facilitated and supported by institutional residents. All members of the institutional campus (residents, children, security staff) should understand the need to perform stray dog feeding only at the designated site.

### Step III and IV of Intervention Mapping: Selecting theoretical model-based intervention method and developing an intervention model

Step III of Intervention Mapping consists of identifying a theoretical model to facilitate behavior change among the target population. To overcome the challenges of provoked dog bites due to lack of understanding of dog gestures, incomplete understanding of the stray dog vaccination schedule, and need for regulated feeding, the Theory of Planned Behavior and Social Cognitive Theory were selected. Based on the theoretical models, a community engagement session was planned. The first component was to target uptake of canine rabies vaccination by providing comprehensive information about its significance and detailing the need for routine immunization to maintain vaccination status. The second component was to address provoked dog bites for which information of dog gestures and prevention of dog bites was given.

Stakeholders in the multidisciplinary planning group were also involved in generating possible measures to be taken to achieve adequate coverage of canine rabies vaccination and stray dog population control. The planning group conceptualized that support from community members is essential to achieve desired vaccination coverage. To ensure continuity of the canine rabies vaccination, collaboration with stakeholders of the veterinary hospital and Non-Government Organization involved in vaccine procurement was necessary. They also highlighted that Institutional administration involvement was essential to conduct sterilization activities to achieve dog population control. The planning group added that the concern of “elimination” of the dog population should also be addressed and neutering of the dogs should be optional. Bridging the gap between the institutional residents was pointed out as an essential need which could be attained by conducting frequent discussions, resolving challenges, and facilitating the activities.

**Fig 3:**
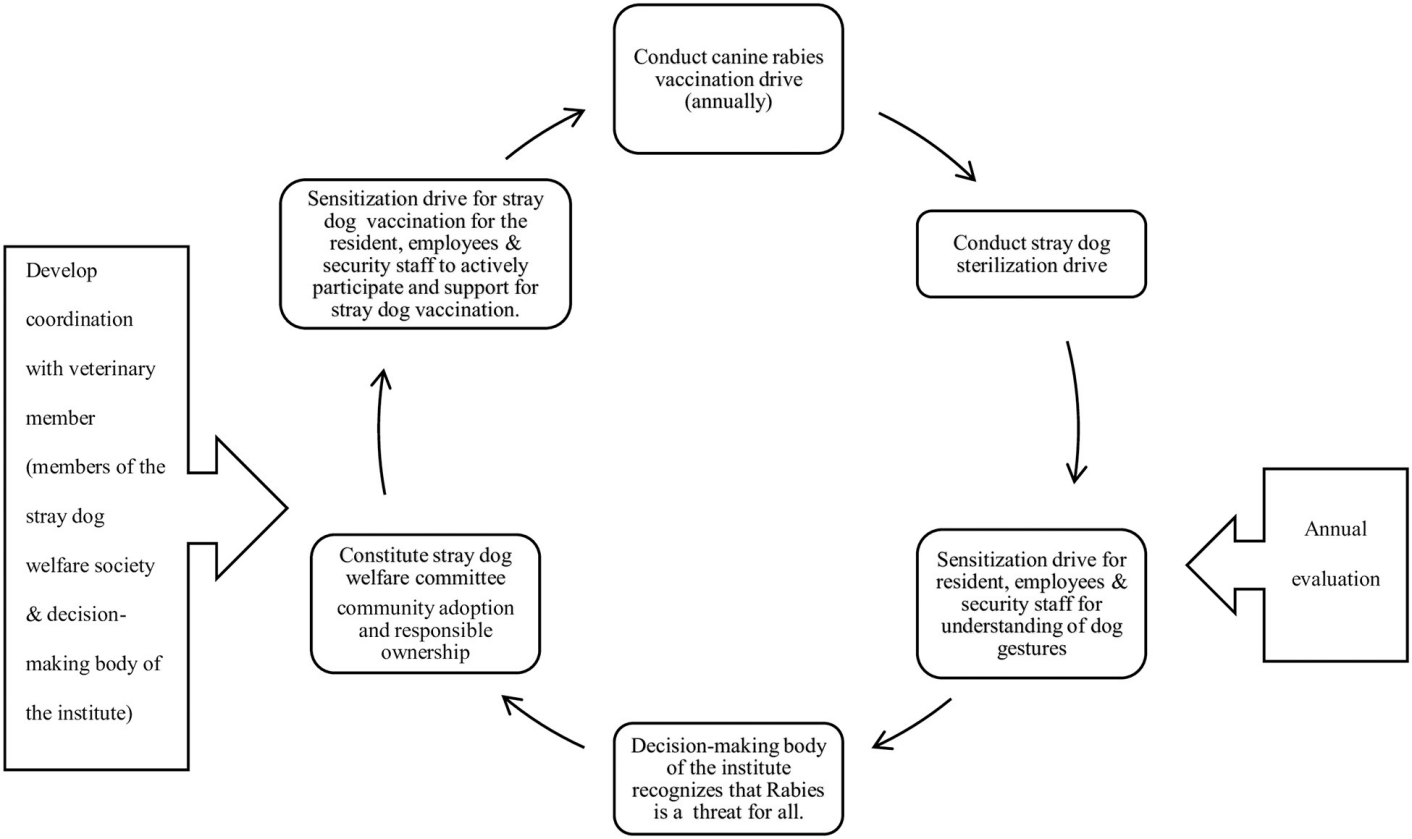
Intervention model. Flowchart representing the intervention model.

Thus based on the recommendations of the core planning group, the intervention model was planned. It consisted of a collaboration between community members and the veterinary sector (to conduct canine rabies vaccination), between the veterinary and non-government organizations to facilitate the supply of canine rabies vaccine, and between the health and veterinary sectors to conduct sterilization activity for dogs (by the provision of Ketamine Hydrochloride). The intervention model so prepared is depicted in Fig 3.

### Step V and VI of Intervention Mapping: Implementation and evaluation

The model so developed was discussed with Institute officials and implemented at the Institutional level. Following measures were conducted.

**Fig 4:**
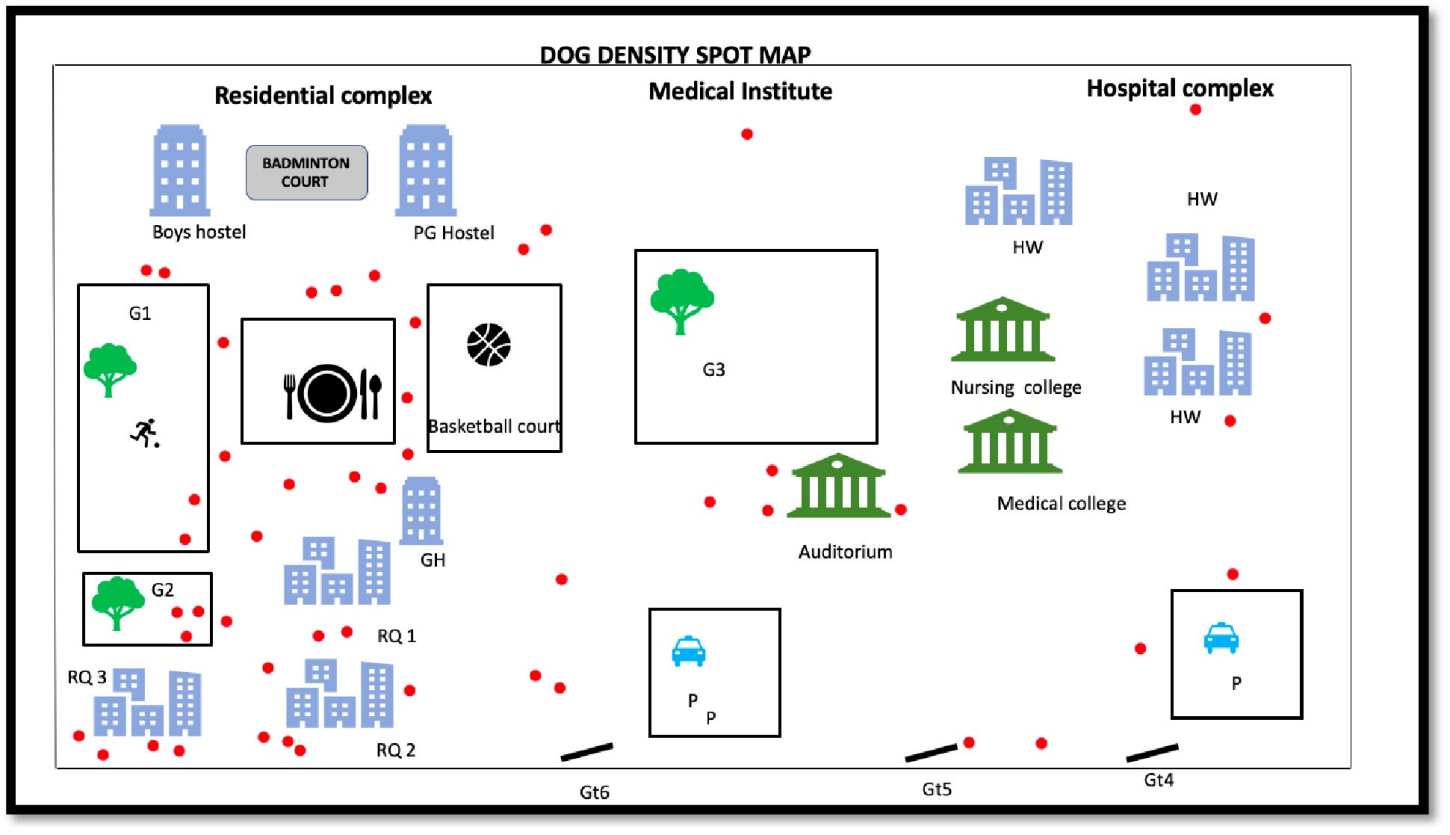
Stray dog density map for the Institutional campus. The spot density map of the institution indicates the concentration of the stray dogs within the campus. Each red dot indicates a stray dog. Gates 1–3 are closed hence not shown in the figure remaining gate i.e. Gate 4–6 are represented by Gt 4–6. Residential complex includes three buildings represented by RQ 1–3; each building has 4 floors. Residential complex has hostel facility for students indicated by Post Graduate hostel (PG), Girls’ Hostel (GH) and Boys’ Hostel. Parking ground (reserved for staff; P). HW indicates Hospital Wing.

First and foremost, campus stray dog density map was prepared. Shown in Fig 4.

### Preliminary results of intervention

It was estimated that there were 46 stray dogs within the campus. Of the total stray dog population, 12 (26.0%) were female dogs. Surveillance for the prevention of rabies has now been strengthened by including campus stray dog bite registry.

Caregivers of the campus stray dogs were sensitized and motivated to participate in vaccination and sterilization of their known stray dogs. Further, after participation in the multidisciplinary planning group, the veterinary service providers were approached to facilitate campus stray dogs’ vaccination. A community-driven stray dog vaccination activity was planned and conducted. During this activity, 35 (76.0%) campus stray dogs were vaccinated.

During the multidisciplinary planning group, it was observed that the supply of Ketamine Hydrochloride was interrupted due to which sterilization of campus stray dogs could not be carried out. Following this, the Institute hospital administration was approached. Subsequently, it was concurred that there is a need to collaborate with the veterinary department to facilitate the successful vaccination and sterilization activities to achieve canine rabies controlled zone. Thereafter, Ketamine Hydrochloride was released for sterilization of campus stray dogs. The residents worked in partnership with one another, hospital administration, and veterinary services department. After ensuring availability of functioning Operation Theatre, 10 (29.4%) male dogs and 7 (58.3%) female dogs were sterilized.

Prior to implementation of the intervention there was no definitive number available for total stray dog population and no official documentation was available for canine rabies vaccination. However, as stated by caregiver of the stray dogs and later confirmed by the representatives from veterinary department, that 10 dogs were vaccinated, thus, showing an increase of 54.3% after intervention. No records were available for sterilization activity. Further, 141 dog bite cases were recorded during the study period, showing a decline [278 cases observed in 2017–2018].

The human resources issue became the rate-limiting step for completion of vaccination of the entire dog population. Only a limited number of veterinary doctors were available who were also involved in multiple zoonotic control activities. However, these dogs were identified for subsequent vaccination round. Further, with regard to the sterilization of dog, the community member had the belief that “*The dog should be able to breed at least once in his/her lifetime and not immediately neutered*”

Community sensitization activity: Health education session on dog behavior, do’s, and don’ts during an encounter with an unknown stray dog was conducted for children residing in the campus. A similar session was also conducted for security staff members and willing members of the residential complex who also were imparted with training to improve their skill to capture a rabid dog in case the need arose.

Following activities have been proposed: -

1. Campus stray dog welfare committee: It was concurred by the participants that stray dogs form a vital part of the natural ecosystem and their (dogs) interest should be protected at all times. Thus, a sustainable solution was proposed i.e. to have a dog welfare committee. Desired role of dog welfare committee is:
  - Supporting the estimation and labelling of the stray dog population of the campus.
  - Actively engage and facilitate annual vaccination and humane sterilization of campus stray dogs.
  - Encourage the residents to feed the stray dog at the designated area(s) within the campus.
  - Encourage the adoption of campus stray dog(s), within the rules and regulation as defined by the residential campus authorities and embracing the operational definition of adoption of community stray dog i.e. taking responsibility of feeding, vaccination, and sterilization of the stray dogs.
2. Responsible stray dog feeding: Responsible feeding would imply having designated and pre-determined feeding sites and timings away from residential buildings and playgrounds. To ensure the practice of responsible dog feeding, it is proposed to impose a fine to those individuals who are feeding the dogs at a non-designated site.
3. Identifiers for vaccinated campus stray dogs: Vaccinated dog will be tagged to ensure identification of vaccinated and non-vaccinated stray dogs.
4. Measure will be taken for prevent the entry of new dog into the campus by closing the gates and vigilantly guard them, by the security staff members.
5. Measure will be taken to ensure that not only the residential campus but also the hospital premises are free of stray dogs’ menace.
6. Integrated forum will be established where activities conducted by the dog welfare committee would be shared with other residents of the complex.

The intervention model so developed was presented to the Institutional administrative head, which is vital to ensure sustainability within the institute. Further, the interventional model was developed not only after taking insights from the institutional residents but also addressing their concerns. This initiated a healthy dialogue between the residents and thus effectuated a canine vaccination activity based on the community’s felt need and facilitated by the community. The benefit of inculcating a felt need was that the canine vaccination was not driven solely by the passive participation of veterinary sectors. Cooperation was also achieved between the government veterinary sector and non-government organizations to overcome any shortage of resources. Further, dog population growth was also a concern, so sterilization activity was conducted where the challenge stated by veterinary stakeholders (shortage of Ketamine Hydrochloride) was addressed by a partnership with the institution (health sector) and the veterinary sector.

## Discussion

This study was conducted with the aim of developing intervention model to design a canine rabies controlled zone using Intervention Mapping framework. Utilisation of Intervention Mapping framework led to holistic assessment of the challenges faced by the residents of the institution and by the veterinary services providers which in turn formed the basis for development of community driven intervention strategy.

Findings from the literature review and in-depth semi-structured discussion with the multidisciplinary planning group revealed that canine vaccination needs prioritization to achieve canine rabies controlled zone. Effectiveness of canine vaccination in elimination of rabies has been documented by various studies [10,12,30–32]. Canine vaccination is essential and needs strengthening. This has been emphasized upon in studies, similar to as seen in the current study [33,34]. Increasing stray dog population and stray dog bite cases were perceived as public threat and was a matter of concern. Similar finding have been reported by authors [28,29]. Multiple print media reports suggests that stray dog population is perceived to be a threat and individual who engage in providing care for stray dog population are commonly treated with hostility [35–37]. In the current study, for the first time a healthy dialogue was achieved between the caregivers and non-caregivers of the stray dogs. Furthermore, a consensus was also attained on the need for conducting stray dog vaccination, sterilization, and regulated stray dog feeding.

It was observed that participants who were involved in stray dog vaccination had incomplete knowledge about routine immunization and the vaccination coverage prior to intervention was below the recommended level. Incomplete vaccination, poor vaccination coverage and lack of awareness about canine vaccine schedule have been reported in Indian settings [13,28,33,38].

Responsible ownership by care givers of stray dogs was emphasised upon in multidisciplinary discussion group. In a review to explore the relationship between general population and street dogs in India, the authors stated that the essential and much-needed role of responsible ownership of dogs was the essence of protecting animal interest [29].

Stray dog feeding has been identified as a common practice, nationally [29,39]. Due to a lack of ownership of the stray dogs, they rely on garbage for food resources. Print media, on multiple occasions, highlight the ongoing, unrestricted stray dog feeding practices. In addition to these findings, an important finding emerged from the current study. There was a felt need for dedicated and timely feeding for stray dogs to protect their (dogs) interest. It is an important finding as timely and correct feeding practices of stray dogs would decrease the occurrence of provoked bites.

During the In-Depth Discussion it was revealed that not all cases of dog bites were unprovoked. Lack of understanding of dog gestures, which could have led to unprovoked dog bites, were previously reported especially among children [29,40,41]. Therefore, reinforcing findings from the current study. This concern was taken up and addressed in the community sensitization activity and subsequently a decrease in number of dog bite cases occurred. It is important to note here that all dog bite cases may not be from a rabid animal, but because rabies is endemic in the canine population, every dog bite case is a potential risk. Thus, it would be worthwhile to reduce the burden of dog bite cases.

The need for collaboration between the veterinary sector, community member, and health sector was also felt. Such collaboration is recommended by WHO but only aspired for in Indian settings [42,43]. Further, we focused on working on the felt need for canine vaccination, which is imperative for its acceptance [43,44]. Mass vaccination of dog in India are driven by Non-Governmental Organisations and dependent on trust based funding [45–47]. Here we present the results where community members facilitated and actively contributed to conduct canine rabies vaccination and sterilization.

Despite availability of ABC program at Jodhpur and previously reported success [24,48], recent statistics shows stray dog population (2012) to be 76,387, showing and alarming increase of over 60% since 2005. [49]. There is limited literature available exploring challenges to effectively implement ABC program in Jodhpur. However, literature from different states reports similar finding where ABC-ARV activities are haphazard due to logistical constraints [29]. Current study highlights few such challenges (lack of resources and infrastructure challenges). Similar findings have been reported in previous surveys conducted to determine the functioning of ABC-ARV program [29,50].

The strength of the study lies in involvement of various stakeholders in intervention model decision making process which is essential to ensure feasibility of any health-related intervention [16]. The study highlights importance of collaborative intervention, driven by community members and supported by health and veterinary stakeholders, is successful in achieving an improvement in vaccination coverage rate, sterilization rate, and a decrease in the number of dog bite cases. Further, Intervention Mapping was utilized to develop the intervention model which is an evidence-based method that guides the development of the program holistically. However, we present the findings from a single institution with a limited number of stray dogs. But, as the Institutional head was involved along with various stakeholder; health, veterinary, administrative sectors and community members participated; we addressed the concern for sustainability.

## Conclusion

Results from needs assessment shows that stakeholder are engaging in canine rabies control activities but the efforts are fragmented. It is imperative to focus on achieving desired vaccination coverage and control of stray dog population by involving the community members to eliminated rabies by 2030. Effective partnership between veterinary sectors and private sector along with collaboration with the health sector would be needed to overcome challenges of logistical concerns. There is scarcity of resources and skill-based training to effectively perform responsible stray dog care. Thus, with effective partnership, and with involvement community members, community members can also be supported on concept of responsible dog ownership. The intervention strategy, when rigorously and in a phase-wise manner along with intersectoral coordination (with veterinary service providers), would set path for achieving a canine rabies controlled zone.

## Supporting information

S1 File Interview guide used for semi-structured interviews. (DOCX)
