# Supplementary material for: Multisectoral approach to achieve canine rabies controlled zone using Intervention Mapping: Preliminary results
Source: PLoS One. 2020 Dec 1;15(12):e0242937. doi: 10.1371/journal.pone.0242937 (PMC7707495; doi:10.1371/journal.pone.0242937)
Supplement: S1 File — (DOCX) [file pone.0242937.s001.docx]

**S1 File**

**Interview guide**

1. Is rabies a concern for your Institution? If so, how. Please share your experience.
2. Do you think that there is a need to address the matter of preventing rabies in your Institution? Explain with reasons.
3. What are the primary prevention strategies you know of apart from post-exposure prophylaxis in humans? Please elaborate.
4. Have you ever participated in canine vaccination activity? Did you face any challenge? Please elaborate.
5. Does your Institution have a framework in place for the prevention of rabies, both in human and canine rabies? According to you, is there any need to focus on canine rabies?
6. According to you, how can we overcome the challenges mentioned earlier?
7. How can the Institution contribute to address your concern for the prevention of canine rabies in the Institution?
8. You, as a member of the veterinary sectors, can you provide detail upon the strategies/ methods adopted by your organization to conduct canine vaccination? Do you face any challenges? Please feel free to share your experience.

Please note no answer is right or wrong. We intend to support the activity conducted by you and want to explore any way that we can support it.

1. You, as a member of the veterinary sectors, can you provide detail upon the strategies/ methods adopted by your organization to conduct animal birth control? Do you face any challenges? Please feel free to share your experience.

Please note no answer is right or wrong. We intend to support the activity conducted by you and want to explore any way that we can support it.

1. What is the compliance or participation of the community in canine vaccination or ABC? Do you think that there is a need for behavior change among the community members to improve their participation in these activities? What are the reasons for this belief?
2. According to you, how can we improve the canine rabies vaccine coverage in the Institution? Please share your experience and give suggestions.
3. According to your experience of conducting canine rabies vaccination and animal birth control activity, what measures need to adopted to ensure the successful implementation of these activities in the Institution?
4. What the challenges you anticipate during canine vaccination activity and ABC activities? Please feel to answer. We intend to ask this question so as to anticipate the challenges and prepare an action plan in advance.
5. Can you suggest some strategies in your experience working in the veterinary sector regarding how these challenges can be overcome?
6. Can our Institution provide support to your activity? If you how?
